# Aesthetic and Functional Rehabilitation of the Primary Dentition Affected by Amelogenesis Imperfecta

**DOI:** 10.1155/2015/790890

**Published:** 2015-02-02

**Authors:** Maria Carolina Salomé Marquezin, Bruna Raquel Zancopé, Larissa Ferreira Pacheco, Maria Beatriz Duarte Gavião, Fernanda Miori Pascon

**Affiliations:** Department of Pediatric Dentistry, Piracicaba Dental School, University of Campinas (UNICAMP), 13414-903 Piracicaba, SP, Brazil

## Abstract

The objective of this case report was to describe the oral rehabilitation of a five-year-old boy patient diagnosed with amelogenesis imperfecta (AI) in the primary dentition. AI is a group of hereditary disorders that affects the enamel structure. The patient was brought to the dental clinic complaining of tooth hypersensitivity during meals. The medical history and clinical examination were used to arrive at the diagnosis of AI. The treatment was oral rehabilitation of the primary molars with stainless steel crowns and resin-filled celluloid forms. The main objectives of the selected treatment were to enhance the esthetics, restore masticatory function, and eliminate the teeth sensitivity. The child was monitored in the pediatric dentistry clinic at four-month intervals until the mixed dentition stage. Treatment not only restored function and esthetic, but also showed a positive psychological impact and thereby improved perceived quality of life. The preventive, psychological, and curative measures of a young child with AI were successful. This result can encourage the clinicians to seek a cost-effective technique such as stainless steel crowns, and resin-filled celluloid forms to reestablish the oral functions and improve the child's psychosocial development.

## 1. Introduction

Tooth enamel is the most highly mineralized structure in the human body, with 85% of its volume occupied by hydroxyapatite crystals [1, 2]. The physiological function and physical properties of enamel are directly related to the composition, orientation, morphology, disposition, and the mineral components within the tissue [3]. During organogenesis, the enamel transitions from a soft and pliable tissue to its final form that is almost entirely devoid of protein [4]. The final composition of enamel is a reflection of the unique molecular and cellular activities that take place during its organogenesis. Deviation from this pattern may lead to amelogenesis imperfecta (AI) [5].

AI is caused by mutations in genes that control amelogenesis and follows inheritance patterns of autosomal dominant, autosomal recessive, or X-linked transmission modes [6–8]. According to Witkop Jr. [9], AI can be classified as hypomaturation, hypoplastic, hypocalcified, and hypomaturation-hypoplastic with taurodontism. In hypomaturation AI, the clinical crowns show normal size and contact with adjacent teeth, but the mottled, brown-yellow enamel is soft. In hypoplastic AI, the teeth are yellowish brown in color, rough in texture, and widely spaced. In hypocalcified AI, the enamel layer may be of normal thickness but is rough and soft and wears away quickly following tooth eruption. In hypomaturation-hypoplastic AI with taurodontism, the enamel is mottled white-yellow-brown in color and is thin at the areas of hypomaturation.

The incidence of AI ranges from 1 : 718 to 1 : 14,000 depending on the population studied [9, 10]. Diagnosis involves the exclusion of extrinsic environment, establishment of a likely inheritance pattern, phenotype recognition, and correlation with the dates of tooth formation to exclude a chronological developmental disturbance [11, 12]. Treatment of AI depends on the individual's specific diagnosis and phenotype. The literature has presented different strategies including the use of glass ionomer cements, composite resin, stainless steel crowns, lab-fabricated crowns, and even multiple extractions necessitating an overdenture [13–16].

Often these AI patients present difficulty in maintaining oral hygiene, decreased masticatory function, and a lower self-esteem, which significantly affect their overall quality of life [17, 18].

The aim of this clinical report was to describe the early treatment in a child with hypocalcified type AI.

## 2. Case Presentation

**Figure 1:**
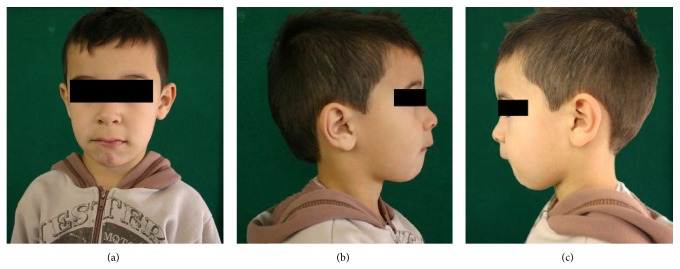
(a) Frontal facial view of a 5-year-old child patient; (b) right lateral facial view; (c) left lateral facial view. Note that patient presented lower lip interposition.

A 5-year-old boy was referred to Department of Pediatric Dentistry at the Piracicaba Dental School, University of Campinas, Piracicaba, SP, Brazil (Figures 1(a)-1(b)), with the complaint of teeth hypersensitivity during meals and presence of yellow teeth. His parents reported that no one in the family has this change. Clinical examination showed generalized tooth wear, as well as enamel loss affecting the entire primary dentition, with the exception of some cervical areas, and poor oral hygiene.

**Figure 2:**
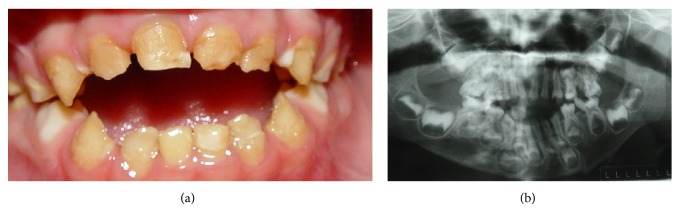
(a) Frontal view of amelogenesis imperfecta; (b) panoramic radiography before treatment.

A panoramic radiographic examination was performed and the loss of primary teeth structure was observed (Figure 2). Based on the clinical and radiographic examination and on the exclusion of other possible etiologic factors, such as syndromes, dental fluorosis, or acquired enamel defects, the AI was classified as hypocalcified type. Also, the child presented pacifier use and bottle sucking, lower lip interposition, and mouth breathe. The clinical characteristics observed were abnormal phonation, anterior open bite, and loss of vertical dimension. In addition, the child presented noncooperation for the treatment.

**Figure 3:**
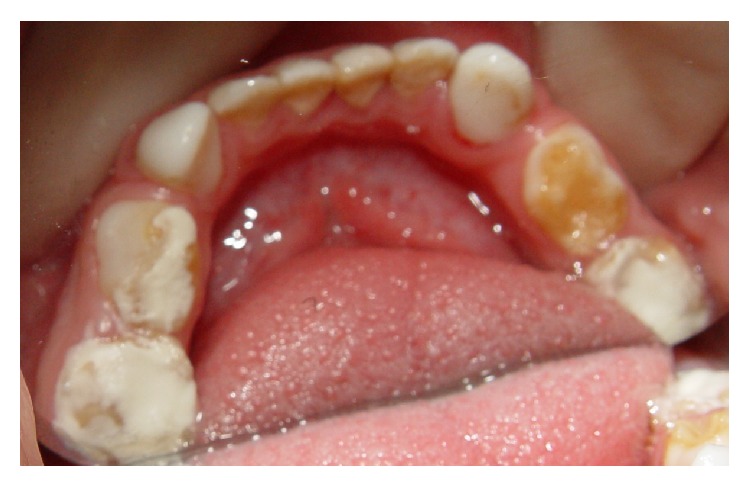
Glass ionomer cement on the mandibular molars in occlusal view.

The treatment of choice was oral rehabilitation with resin-filled celluloid forms for upper and lower primary incisors and canines and stainless steel crowns on the primary molars. The child's family was instructed about the oral hygiene and the oral habits and management childhood behavior was also realized. A questionnaire on daily food intake was answered by the parents and information about good feeding and eating habits was provided, since the practiced type of nutrition has a direct impact on oral and overall health; moreover, an adequate food, with a balanced intake of nutrients necessary for the proper functioning of the body, is critical to the child's growth and development, contributing to the success of treatment [19–21]. The initial objective during the treatment was to alleviate the dentinal teeth hypersensitivity, which was achieved by placing glass ionomer cement on the mandibular molars and fluoride varnish application on the anterior teeth (Figure 3). The benefit of fluoride released from glass ionomers is to promote additional preventive effects, not only acting in the dental structure resistance, but also controlling the growth of* S. mutans* [22, 23]. Also, dentin bonding agents seek to obliterate the dentinal tubules, reducing or ceasing hypersensitivity.

The restoration of the maxillary and mandibular primary incisors and canines was performed using composite resin-filled celluloid forms (TDV Dental, Pomerode, SC, Brazil) in accordance with the manufacturer's instructions. The procedures were as follows: prepare the forms so they fit directly over the teeth; perforate the lingual surface to overflow the composite resin; isolate the teeth with cotton rolls; acid-etch for 30 seconds with phosphoric acid at 37% (3M/ESPE, St. Paul, MN, USA); rinse with water; change the cotton rolls; gently air-dry; apply two layers of adhesive system (Single Bond, 3M/ESPE, St. Paul, MN, USA) and light-cure following the manufacturer's instructions; apply a light-cured microhybrid resin-composite Opallis (Shade A 0,5/FGM, Joinville, SC, Brazil) inside the celluloid forms; seat the forms with finger pressure; remove material excess; light-cure the restorations; remove the outer forms; check the occlusion; and then finish and polish.

**Figure 4:**
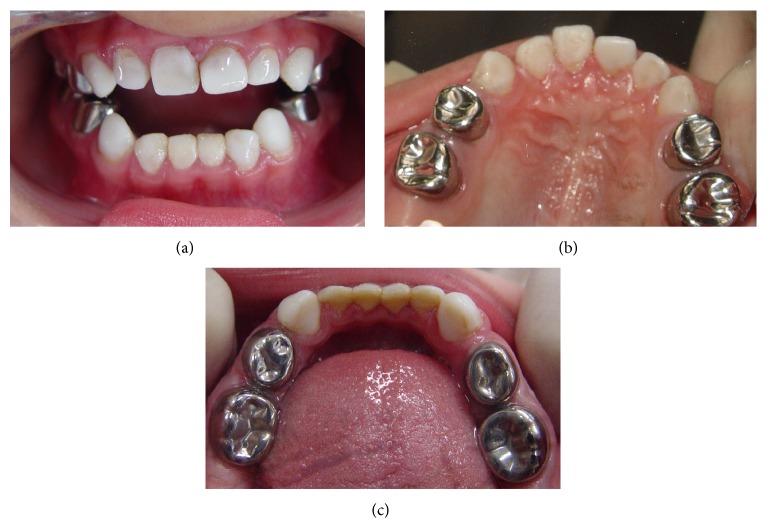
(a) Frontal view of the completed treatment with stainless steel crowns and composite resin-filled celluloid forms in a 5-year-old child; (b) maxillary occlusal view; (c) mandibular occlusal view.

The stainless steel crowns were selected and the adjustments were made with carborundum disc (Saint-Gobain Abrasivos Ltd, Carapicuiba, SP, Brazil), with #114 pliers to the satisfactory fit and rubber abrasive points (KG Sorensen, São Paulo, SP, Brazil) to the polish. The interproximal and occlusal tooth surfaces were prepared with diamond burs (KG Sorensen, São Paulo, SP, Brazil). Glass ionomer cement (Vidrion R, SS. White, Rio de Janeiro, RJ, Brazil) was used according to the manufacturer's instructions and placed inside the crowns. Firm seating pressure was exerted on each crown using finger pressure alone. The patient's vertical dimension of occlusion was reestablished with the aid of the stainless steel crowns (Figure 4).

Since the mother reported that the child had trouble breathing at night with snoring, he was forwarded to the otolaryngologist. Adenotonsillar hypertrophy was diagnosed and then tonsil removal surgery was carried out. After that, the mother reported that the child had improved breathing. He was forwarded to the speech therapist due to speech difficulties and muscular hypotonia. The child has been recalled for the preventive maintenance at four-month intervals. It is expected that permanent teeth should present AI also. Along the follow-up, the respective diagnosis will be performed and the measurements regarding dietary counseling and instruction about the oral hygiene will be maintained, as well as the treatment of dentinal hypersensitivity, if necessary. Furthermore, after complete eruption of permanent teeth, aesthetic and functional rehabilitation will be performed, if indicated.

## 3. Discussion

AI is a developmental, often inherited disorder, affecting the primary and permanent dentition. It usually occurs in the absence of systemic features and comprises diverse phenotypic entities [4]. The extensive rehabilitation of a young patient with a generalized AI is a challenge for the clinician, and a multidisciplinary team of professionals needs to be involved in the care plan [12].

Previous studies have reported several methods for determining AI type using combinations of clinical, radiographic, histologic, and genetic criteria [24, 25]. The AI type in this case report was classified according to the clinical features (phenotypes) and radiographic images [26–28]. The chalky dull color of the enamel represents low mineralization, clinically expressed by pigmented, softened, and easily detachable enamel structure and this aided in the diagnosis of hypocalcified AI.

Children with AI can have high dental needs and may present many dental challenges. Several factors have to be taken into consideration, including the age of the patient, the quality and quantity of existing enamel and tooth structure, the periodontal condition, and the long-term prognosis and stability of the result. The multiple treatment phases often last several years, and at each stage, the long-term consequences, risks, and benefits of the various therapy options must be discussed with patients and parents [29]. The successful management of AI during childhood requires the cooperation and motivation of the patient and parents [30]. In this case report, there was cooperation from the patient and his parents, determining the effectiveness of treatment.

Historically, patients with severe AI have been treated with multiple extractions of the primary teeth followed by the construction of complete dentures [31]. Nowadays, there is a range of materials used to restore the teeth that includes the use of composite resin, stainless steel crowns, glass ionomer cement, and functional maintainer appliance [31, 32]. The objective during the first phase of the treatment was to relieve teeth hypersensitivity of the patient, which was achieved by placing glass ionomer cement on the mandibular and maxillary molars. According to the mother, after the restorative procedures, the patient's dental hypersensitivity disappeared completely and the feeding could be improved.

Attention should first be given to the patient's level of oral hygiene and dietary habits, which can compromise the rehabilitation procedures. Poor oral hygiene is a recognized problem in patients with AI, due to the rough enamel surface which causes biofilm retention and the sensitivity experienced when brushing [33]. Therefore, oral hygiene instructions are essential for the successful treatment [32].

In children with the primary and the early mixed dentition, stainless steel crowns are an effective type of restoration in managing tooth sensitivity and restoring severely broken down primary molars and permanent molars in children [13, 17]. The stainless steel crowns are extremely durable, relatively cheap, and subject to minimal technique sensitivity during placement and offer the advantage of full coronal coverage. In primary teeth, the stainless steel crowns are indicated following pulpotomy/pulpectomy and are also applicable for teeth with developmental defects, large carious lesions involving multiple surfaces where amalgam is likely to fail and fracture teeth [34, 35]. Despite being unattractive, stainless steel crowns in posterior teeth do not compromise the aesthetic; the time consuming to fit is compensated by the fact that one session is necessary to finish them and the adequate practice on manipulation can guarantee successful restorations, associated with oral hygiene measurements.

In the present case, due to age of the patient and considering the longevity of the maxillary and mandibular primary molars in the arches (approximately six years), stainless steel crowns were placed [36]. These crowns are able to reproduce the anatomy of primary molars with accuracy, considering the contour and occlusal surface. The crowns are precrimped at the cervical margin to provide good retention and snap fit [29]. Considering all these factors, stainless steel crowns were the treatment of choice for the posterior teeth rehabilitation.

With respect to the primary anterior teeth restorations, advances in esthetic dentistry, particularly in bonding to dentin, allowed for the reestablishment of function and acceptable esthetics, especially with respect to the short primary teeth cycle [37, 38]. Composite resin restorations have been advocated to mask discoloration and improve dental esthetics. Composite resin restorations can be placed with minimal or no tooth preparation to preserve tooth structure and provided satisfactory esthetics. In addition, composite resin restorations were clinically successful in children with hypocalcified AI during 36 months of follow-up [39]. In the same context, the use of resin-filled celluloid forms in the maxillary and mandibular primary anterior teeth was the most suitable treatment for this 5-year-old boy. A recent retrospective study shows the need for long-lasting restorative solutions for patients with AI. It also shows the importance of establishing an early permanent therapy plan for these patients to avoid frequent dental visits [40].

In addition, AI has a significant impact of the psychosocial development on the affected individuals [18]. When the related patient first arrived in the pediatric dentistry clinic, he was very shy, he had tooth hypersensibility during meals which made it difficult for him to eat well, and these factors resulted in inappropriate psychological behavior. A substantial improvement was soon observed in his eating habits, according to his parents.

Feeding and esthetic expectations of the patient were achieved. In the clinical examination, no problem was seen in soft tissue or in maintenance of the restorations. However, it should be emphasized that regular follow-up is extremely important for the long-term maintenance of this complex treatment.

## 4. Summary

Preventive aspects in the primary dentition include dietary advice, oral hygiene instructions, and topical fluoride application. Treatment planning for patients with AI is related to many factors: the age and socioeconomic status of the patient, the type and severity of the disorder, and the intraoral situation at the time the treatment is planned. It includes removal of surface stains, reducing sensitivity, maintaining vertical dimension of occlusion, and the esthetics with adhesive restorations. This paper is an attempt to improve the clinician's knowledge about the clinical diagnosis as well as intervention required for such a condition.

## Clinical Significance

The preventive, psychological, and curative measures of a young child with AI were successful. This result can encourage the clinicians to seek a cost-effective technique such as stainless steel crowns and resin-filled celluloid forms to reestablish the oral functions and improve the child's psychosocial development.
